# Liver fibrosis causes periportalization of lobular zonation

**DOI:** 10.17179/excli2019-2078

**Published:** 2019-12-20

**Authors:** Abdellatief Seddek

**Affiliations:** 1 Department of Forensic Medicine and Toxicology, Faculty of Veterinary Medicine, South Valley University, Qena, Egypt

## ⁯⁯⁯⁯⁯⁯⁯⁯

The human liver consists of approximately one million liver lobules, which are known to show metabolic zonation (Braeuning et al., 2006[3]; Halpern et al., 2017[11]; Saito et al., 2013[20]). Zonation is the spatial separation of different metabolic pathways along the porto-central axis of the liver lobule (Gebhardt and Matz-Soja, 2014[5]; Kietzmann, 2019[17]; Godoy et al., 2013[9]). For example, many phase-I-metabolizing enzymes are located in the center of the liver lobule (Schenk et al., 2017[21]; Sezgin et al., 2018[23]; Ghallab, 2017[6]). The advantage of this arrangement is that many xenobiotics are detoxified before they are drained into the central vein and reach the general circulation (Hewitt et al., 2007[14]; Bartl et al., 2015[1]; Schliess et al., 2014[22]). However, some compounds are metabolically activated by pericentrally expressed liver enzymes (Gebhardt et al., 2003[4]; Bolt, 2017[2]; Hengstler et al., 2000[13]). This leads to a pericentral pattern of necrosis induced by many hepatotoxic compounds that require metabolic activation (Hammad et al., 2017[12]; Hoehme et al., 2007[16]; 2010[15]). 

Liver fibrosis is caused by chronic liver damage that leads to inflammation and scarring (Pimpin et al., 2018[19]; Weiskirchen and Tacke, 2016[24]; Gressner and Weiskirchen, 2006[10]; Leist et al., 2017[18]). Currently, little is known how liver fibrosis influences lobular zonation. In a recent issue of Cells, a study has been published, demonstrating that liver fibrosis causes 'periportalization' of lobular zonation (Ghallab et al., 2019[8]). Periportalization means that the entire liver lobule adopts a periportal gene expression pattern, including the pericentral zone. To study this phenomenon, RNA-sequencing data were generated using fibrotic livers of mice caused by repeated CCl_4 _administration (Ghallab et al., 2019[8]). Interestingly, pericentral genes were enriched among genes downregulated by CCl_4_, while periportal genes were enriched among the upregulated genes. This pattern of periportalization was confirmed by immunostaining. It also occurred when liver fibrosis was induced by a mouse model of obstructive cholestasis (Ghallab et al., 2019[8]). The advantage of a periportalized lobular zonation is that hepatotoxic xenobiotics that require metabolic activation by cytochrome P450 enzymes cause less damage to the liver. This has been shown by the authors using the example of acetaminophen (Ghallab et al., 2019[8]). However, this advantage is obtained at the expense of suboptimal fine-tuning of physiological metabolic functions, e.g. detoxification of ammonia (Ghallab et al., 2016[7]). It will be interesting to learn in future, whether periportalization of lobular zonation demonstrated in fibrotic mouse livers also occurs in human liver fibrosis. 

## Conflict of interest

The author declares no conflict of interest.
